# Development and Usability of an Advance Care Planning Website (My Voice) to Empower Patients With Heart Failure and Their Caregivers: Mixed Methods Study

**DOI:** 10.2196/60117

**Published:** 2024-12-18

**Authors:** Chetna Malhotra, Alethea Yee, Chandrika Ramakrishnan, Sanam Naraindas Kaurani, Ivy Chua, Joshua R Lakin, David Sim, Iswaree Balakrishnan, Vera Goh Jin Ling, Huang Weiliang, Lee Fong Ling, Kathryn I Pollak

**Affiliations:** 1 Lien Centre for Palliative Care, Duke-NUS Medical School, 8 College Road, Singapore, 169857, Singapore, 65 65165692, 65 62217372; 2 Program in Health Services and Systems Research, Duke-NUS Medical School, Singapore, Singapore; 3 Department of Supportive and Palliative Care, National Cancer Centre Singapore, Singapore, Singapore; 4 Psychosocial Oncology and Palliative Care, Dana Faber Cancer Institute, Boston, MA, United States; 5 Department of Cardiology, National Heart Centre Singapore, Singapore, Singapore; 6 Department of Cardiology, Sengkang General Hospital, Singapore, Singapore; 7 Department of Internal Medicine, Singapore General Hospital, Singapore, Singapore; 8 Department of Cardiology, Changi General Hospital, Singapore, Singapore; 9 Department of Cardiology, Khoo Teck Phuat Hospital, Singapore, Singapore; 10 Cancer Prevention and Control, Duke Cancer Institute, Durham, NC, United States; 11 Department of Population Health Sciences, Duke University School of Medicine, Durham, NC, United States

**Keywords:** advance care planning, decision aid, heart, website, heart failure, care plan, caregiver, usability, acceptability

## Abstract

**Background:**

Web-based advance care planning (ACP) interventions offer a promising solution to improve ACP engagement, but none are specifically designed to meet the needs of patients with heart failure and their caregivers.

**Objective:**

We aimed to develop and assess the usability and acceptability of a web-based ACP decision aid called “My Voice,” which is tailored for patients with heart failure and their caregivers.

**Methods:**

This study’s team and advisory board codeveloped the content for both patient and caregiver modules in “My Voice.” Using a mixed methods approach, we iteratively tested usability and acceptability, incorporating feedback from patients, caregivers, and health care professionals (HCPs).

**Results:**

We interviewed 30 participants (11 patients, 9 caregivers, and 10 HCPs). Participants found the website easy to navigate, with simple and clear content facilitating communication of patients’ values and goals. They also appreciated that it allowed them to revisit their care goals periodically. The average System Usability Scale score was 74 (SD 14.8; range: 42.5-95), indicating good usability. Over 80% (8/11) of patients and 87% (7/8) of caregivers rated the website’s acceptability as good or excellent. Additionally, 70% (7/10) of HCPs strongly agreed or agreed with 11 of the 15 items testing the website’s acceptability.

**Conclusions:**

“My Voice” shows promise as a tool for patients with heart failure to initiate and revisit ACP conversations with HCPs and caregivers. We will evaluate its efficacy in improving patient and caregiver outcomes in a randomized controlled trial.

## Introduction

Advance care planning (ACP) is a process to support individuals in understanding and sharing their values, goals, and preferences regarding medical care [1]. Systematic reviews by our team reveal that while ACP may not consistently result in goal-concordant care for patients [2], improve their quality of life, or reduce health care expenditures, it can enhance communication of patient values and goals with health care professionals (HCPs) and caregivers or surrogate decision makers [3,4]. This, in turn, equips patients, caregivers, and HCPs to be better prepared for making in-the-moment health care decisions, emphasizing “preparation” rather than “planning” as an objective for ACP [5]. This emphasis on preparation is crucial, considering that most patients and caregivers are often unprepared for making these decisions [6]. Effective preparation involves educating patients about their illness and enabling them to share their values and goals with their doctors and surrogate decision makers.

Despite these clear benefits, ACP completion rates remain low worldwide [7-12]. This is particularly concerning for patients with conditions such as heart failure, which have an unpredictable clinical trajectory that makes prognostication difficult. This uncertainty in prognosis often leads to delays or avoidance of ACP conversations [13,14]. Furthermore, ACP conversations and documentation require substantial time and effort, often dissuading clinicians from initiating them [15-17]. Patients themselves may lack the readiness, initiative, and knowledge to initiate these conversations [18-21]. Most notably, even when ACP conversations happen, they are conducted as a one-time occurrence rather than as part of an ongoing process [2]. Our previous research has revealed that patients’ care goals change over time, thus limiting the value of one-time ACP conversations, and requiring that ACP conversations be revisited periodically [22-25].

To enhance ACP completion rates among patients with heart failure and foster a truly patient-centered approach, it is crucial to empower patients to initiate ACP conversations with their caregivers and HCPs. ACP web-based decision aids offer a promising solution, preparing patients for these conversations while alleviating the time burden for clinicians. Yet, a scoping review of 11 web-based ACP decision aids for patients identified only two applicable for patients with heart failure, with only one specifically tailored for this group [26,27]. Furthermore, while these decision aids also hold promise for enabling periodic patient-driven revisions and providing access to the latest updates on patients’ evolving care goals, none of the existing decision aids incorporated mechanisms to promote a systematic reconsideration of patients’ care goals. With the exception of few web-based decision aids, most existing ACP decision aids also do not encourage the active involvement of caregivers in the ACP process, and simply coach patients to engage with them [28-33]. The latter is particularly pertinent given the crucial role caregivers play in the decision-making process in many settings including Singapore, where this study is based [34].

To address these gaps, we developed “My Voice,” a web-based ACP decision aid tailored for patients with heart failure and their caregivers. It educates users about their illness, enables sharing and systematic reconsideration of patients’ values and goals, and actively involves caregivers. This paper aims to present the development process, and usability and acceptability of “My Voice” among patients, caregivers, and HCPs. Given the extensive engagement with patient representatives and HCPs during its development, we hypothesize that “My Voice” will meet the standardized System Usability Scale (SUS) score cutoff of 68, as proposed by Lewis and Sauro [35].

## Methods

### Development of ”My Voice”

Between April 2022 and May 2023, we engaged in an extensive process involving literature reviews, examining existing ACP decision aids, and consultations with a study team comprising diverse experts including health services researchers, cardiologists, palliative care physicians, social workers, communication coaches, and information technology professionals. We also established a study advisory board consisting of patient representatives and HCPs trained to conduct ACP conversations and gathered their inputs regarding content and structure for the interactive web-based application “My Voice.” Guided by the COM-B model that focuses on capability (C), opportunity (O), motivation (M) to enhance behaviors (B) [36], “My Voice” aimed to improve patients’ capability for engaging in ACP conversations, creating opportunities for them to have these conversations and revisit them periodically, and motivating them to do so.

A professional production house produced narration-style videos featuring HCPs from this study’s team. The website content and videos were initially developed in English and subsequently professionally translated into two local languages (Mandarin and Malay) to ensure inclusivity and accessibility. Upon completion of the content development phase, we developed the initial prototype of “My Voice.” To ensure the security of participants’ identifiable information, we incorporated password protection and a two-factor authorization process. The research team obtained relevant institutional approvals at all stages of web application development.

### Description of “My Voice”

“My Voice” includes patient and caregiver modules. The patient module consists of a series of educational videos lasting 1-2 minutes each, organized into five steps: (1) learn about heart failure, (2) think about what is important to you, (3) why and how to choose a spokesperson, (4) speak to your doctor about what is important to you, and (5) revisit “My Voice.”

In addition to the videos, step 1 includes a knowledge quiz. Step 2 incorporates questions to elicit patient values and goals (referred to as value clarification questions), which were based on the Serious Illness Conversation Guide [37]. Step 3 provides fields for nominating up to two surrogate decision makers, known locally as the nominated health care spokespersons. Upon completion of step 5, the patients’ responses to the value clarification questions and the details of their spokespersons are automatically populated into a summary document called the “My Voice” document. Patients can view and edit the document before it is saved on the website. The website then triggers automated emails and phone text messages containing the document to both patients and their designated surrogate decision makers. Patients and their designated surrogate decision makers can also view and print the document anytime through the website. The website also sends phone reminders to patients to revisit “My Voice.” Multimedia Appendix 1 highlights select pages from the “My Voice” website.

The caregiver module includes educational videos lasting 1-2 minutes structured into three steps: (1) learn about heart failure, (2) talk to your loved one, and (3) support your loved one. As with the patient module, step 1 incorporates a knowledge quiz. At the end of the module, caregivers have the option to view the patient’s “My Voice” document.

Between May and August 2023, we recruited a convenience sample of patients with heart failure, their caregivers, and HCPs, from four public hospitals in Singapore. These groups of participants are the key stakeholders in ACP conversations, and hence best positioned to provide feedback on the website. We recruited patients from outpatient clinics and wards, based on the following inclusion criteria: (1) adults aged 21 years or older; (2) diagnosed with heart failure; (3) Singaporean or permanent resident; (4) able to understand either English, Mandarin, or Malay; and (5) willing to use a web-based intervention. Caregivers of the above eligible patients were approached independently and included if they consented. Caregivers were: (1) adults aged 21 years or older; (2) providing informal care or ensuring the provision of care, or serving as the main decision maker for the patients with heart failure with no expectation of financial compensation; (3) able to understand either English, Mandarin or Malay; and (4) willing to engage with a web-based intervention. HCPs from department of cardiology namely cardiologists, cardiology nurses, and medical social workers trained in having ACP conversations were included.

### Testing Procedure

We used a mixed methods design incorporating both qualitative interviews that provided feedback to iteratively revise the website’s content and design to improve the overall user experience, and quantitative surveys to estimate the website functionalities including its usability and acceptability. The results from qualitative and quantitative sections were integrated during interpretation as a narrative discussion.

Trained research staff (IC and SNK) conducted usability testing on a tablet device provided by the research team. Patients and caregivers viewed their respective modules while HCPs viewed both modules. On viewing the relevant modules, participants responded to open-ended questions during a qualitative interview and answered a brief questionnaire. Research staff facilitated navigation and ensured participants viewed all steps. All study procedures were completed in one sitting.

### Qualitative Feedback

A topic guide developed by the research team based on concepts from the user-experience model [38]. The guide comprised of open-ended questions eliciting participants’ perceptions and satisfaction with the design and content of the website, and feedback for enhancement. Questions were tailored for each participant group (Multimedia Appendix 2). Participants were given time to explore and navigate each page of the website. Subsequently, they were prompted to provide feedback on the website’s navigational ease, and clarity of on-page explanations for each step. Research staff facilitated website navigation and ensured the participants completed all steps. Specifically, patients and HCPs were probed on the comprehensibility of the value-clarification questions and whether they could easily select their preferred answers from a potential list of response options. Their suggestions for refining the questions and the response options were elicited. Lastly, participants were asked about their overall impressions of the website, its perceived usefulness, aspects they liked most or least, and recommendations for improvement. Participant responses were audio-recorded and transcribed (to English if conducted in Mandarin or Malay) for analysis. The interviews were conducted in a private room within the health care facility and lasted between 30‐80 minutes. We analyzed qualitative data concurrently with data collection. Data saturation was achieved by the 27th interview, when no new feedback about the website emerged.

### Survey

After viewing the respective modules, participants answered a survey in the language participants viewed the website (English, Mandarin, or Malay). The survey collected demographic information, and questions assessing usability and acceptability. Usability was evaluated by the 10-item SUS; each item was rated on a 5-point Likert scale (strongly disagree to strongly agree) [39,40]. We assessed acceptability using the acceptability rating scale developed by the Ottawa Hospital Research Institute adapting it to specific decision-making aspects of our study [41]. Patients and caregivers, rated items on a 4-point Likert scale ranging from poor to excellent (score: 1‐4), and for HCPs used a 5-point Likert scale ranging from strongly disagree to strongly agree (score: 1‐5). Example items included, “it will be easy for me to use ‘My Voice’ for introducing ACP to my patients” and “this ‘My Voice’ website is better than how I usually go about conducting ACP” [41]. Lastly, patients and caregivers rated the length (too long, too short, or just right) and amount of information (too much, too little, or just right) for “My Voice.”

The surveys were first developed in English and then translated by native speakers into Mandarin and Malay and verified by a second team member fluent in the language. Before fielding, the surveys were tested with volunteers from a nonclinical setting.

### Sample Size

Previous literature suggests that a sample of 20 participants can identify 95% of usability problems [42]. Thus, a sample size of 30 was deemed to be sufficient for usability testing. Previous studies assessing usability of digital interventions also had similar sample sizes [43].

### Data Analysis

Two authors CR and IC analyzed the open-ended interview transcripts using qualitative description methodology [44]. We categorized the interview feedback from participants into broad concepts (eg, language or layout) paying close attention to positive and negative views toward the website to derive themes and subthemes inductively. The two authors verified each other’s coding carried out in Excel (Microsoft Corp), discussed and agreed upon the final themes derived. From the survey data we described participants’ demographic and health status characteristics. We also calculated the total SUS score as a sum of each item score and rescaled it within the range of 0 to 100. A higher score signified greater usability, and a score greater than 68 indicated good usability [39,40]. We then present the total scores for each participant group and overall sample. For each item on the acceptability scale, we calculated the proportion of patients and caregivers responding as good or excellent and HCPs rating as agree or strongly agree.

### Ethical Considerations

This study was approved by the SingHealth Centralized Institutional Review Board in Singapore (2022/2482) and was conducted in compliance to the institutional guidelines. Participants were briefed on this study’s purpose and had the option to withdraw at any time. We obtained written informed consent and provided US $20 cash as compensation upon completion of usability testing procedures. All data was deidentified to protect participants' privacy and confidentiality.

## Results

### Participant Characteristics

A total of 30 participants out of 44 (response rate 68%) consented including 11 patients, 9 caregivers, and 10 HCPs. Among them 21 (10 HCPs, 6 patients, and 5 caregivers) reviewed the English version, 7 (4 patients and 3 carergivers) reviewed the Mandarin version, and 2 (1 patient and 1 caregvier) reviewed the Malay version of the website, respectively, and completed the surveys in the respective languages. Participants’ mean age was 49 (SD 14.9) years and 60% (18/30) were females. Participant characteristics are described in Table 1.

**Table 1.** Participant characteristics (N=30).

Item	Patients (n=11)	Caregivers (n=9)	Health care professionals (n=10)
Age (years), mean (SD)	60.7 (13)	44.9 (15.4)	39.8 (6.1)
**Gender, n (%)**			
Male	7 (64)	2 (22)	3 (30)
Female	4 (36)	7 (78)	7 (70)
**Ethnicity, n (%)**			
Chinese	8 (73)	7 (78)	—^a^
Malay	2 (18)	1 (11)	—
Indian	1 (9)	0 (0)	—
Other	0 (0)	1 (11)	—
**Marital status, n (%)**			
Married	8 (73)	6 (67)	—
Widowed	1 (9)	0 (0)	—
Never married	2 (18)	3 (33)	—
**Education, n (%)**			
Secondary school	5 (46)	4 (44)	—
Junior college, polytechnic, diploma, or vocational	2 (18)	2 (22)	—
University and above	4 (36)	3 (34)	—
**Duration of heart failure, n (%)**			
<5 years	5 (45.5)	—	—
5 to <10 years	5 (45.5)	—	—
10 years and above	1 (9)	—	—
**Relationship with patient, n (%)**			
Spouse	—	1 (11)	—
Child (son or daughter)	—	7 (78)	—
Others (relative)	—	1 (11)	—
**Profession, n (%)**			
Cardiologist	—	—	4 (40)
Nurse	—	—	3 (30)
Medical social workers trained in advance care planning	—	—	3 (30)
**Experience with treating patients with heart failure, n (%)**			
Less than 5 years	—	—	1 (10)
5 to <10 years	—	—	5 (50)
10 years and above	—	—	4 (40)
**Advance care planning training, n (%)**			
Yes	—	—	6 (60)
No	—	—	4 (40)

a Not applicable.

### Qualitative Feedback and Iterative Redesign

#### Overview

The following three themes describe participants’ feedback and suggestions, and highlight the revisions made by the research team.

#### Theme 1: User Experience of Navigating the Website

Many participants provided positive feedback about the ease of navigation and layout of the website. Patients, caregivers, and HCPs described the interactive features as easy to use and appreciated the simple layout of the website. However, two HCPs anticipated that older patients may have difficulty reading extensive text due to poor eyesight, scrolling down the web page, and navigating the site.

To address these concerns and enhance user experience and accessibility for older patients, we added a note on the expected time to complete the website and a progress bar to allow users to track their progress. We also increased the frequency of navigational buttons such as the “submit My Voice document,” increased the font size and changed the font color to improve readability. Lastly, within the help section, in addition to allowing the participants to type their queries, we incorporated a drop-down list for them to select from. This enhanced the ease of reaching out to the research team in case of difficulties.

#### Theme 2: Acceptability of Website Content and Duration

Patients, caregivers, and HCPs found the language clear and straightforward with “no jargon.” However, two HCPs suggested reducing the wordiness of the web page. Most participants also found the duration of the website to be suitable although one HCP recommended adding a pause button for patients who may need breaks or prefer to complete the website in smaller segments.

Some patients expressed that the quiz explanations were overly direct and demoralizing. Given the discomfort surrounding the topic of death and dying, they suggested incorporating elements of hope to make the website more comforting.

We received extensive feedback about the value-clarification questions and their response options. One HCP recommended adding details about caregiving arrangements and clarifying terms such as “physically comfortable” or “at peace.” Another HCP highlighted that being dependent on others did not equate to being a burden on the family. Participants also had challenges understanding and responding to a question asking patients’ willingness to trade-off between quality and length of life, despite multiple iterations and revisions. These revisions aimed to clarify the question, prompting patients to imagine a situation where such a trade-off would occur. We also changed the initial 3-point response to a 2-point response option, excluding the choice to prioritize both aspects simultaneously. Participants described this question as vague and difficult to relate to.

In response to these concerns, we shortened the introduction section of the website and reduced the wordiness of each web page. To enable participants to complete the website in multiple sessions, we implemented a feature that displays their previous responses if they have not submitted their “My Voice” document. We also simplified the phrasing of key terms and modified the explanations for the quiz responses to be more emphathic, acknowledging patients’ desire for a cure and emphasizing that symptoms can be managed even though heart failure is incurable. We clarified the response options for the value-clarification questions by making them more specific and split up the initial option of “being dependent on others for their daily activities and being a burden on their family” into two separate response options. We removed the question assessing the trade-off on quality and length of life.

#### Theme 3: Usefulness in Understanding Patient’s Values and Goals

Many HCPs noted that the website could complement and enhance existing ACP processes by helping patients reflect on their values and care goals, thereby preparing them for the challenging in-person ACP conversations ahead of time. This preparation could potentially save time during dedicated clinic appointments for ACP conversations. Patients and caregivers also found the website useful as it encouraged them to communicate with each other. Caregivers particularly appreciated that patients’ “My Voice” document could be updated periodically to reflect their changing care goals.

Table 2 presents example quotes illustrating feedback obtained, and Table 3 outlines participants’ suggestions for improvement alongside the corresponding revisions made.

**Table 2.** Participant feedback on “My Voice” website.

Subtheme		Patients	Caregivers	HCPs^a^	Selective positive quotes
**Theme 1: user experience of navigating the website**					
	Ease of navigation	✓✓×^b^	✓✓✓	✓✓×	“Easy and straightforward. Quite nice to use” [UAT25, patient] “Like most interfaces used in Singapore, it’s not particularly difficult…overall quite easy to use…I am quite sure that they (elderly) will need somebody to go through with them, for the elderly patients” [UAT05, cardiologist]
	Simple layout	✓✓✓	✓✓✓	✓✓✓	“My favourite part is the document that is generated with the spokesperson. It’s written very clearly” [UAT24, caregiver] “Not too cluttered, quite clearly delineated” [UAT05, cardiologist]
**Theme 2: acceptability of website duration and content**					
	Ease of understanding	✓✓✓	✓✓✓	✓✓✓	“Very easy, very simple to understand” [UAT29, patient] “I think the options listed inside are easy to understand…Questions quite straightforward” [UAT01, ACP^c^ facilitator] “Videos did not contain any jargon, so it’s quite clear” [UAT05, cardiologist]
	Informative	✓✓	✓✓✓	✓✓✓	“Very succinct, it tells me exactly what I need to do” [UAT25, patient] “I think it’s good that we have all this background learning, at least we know and can be more educated...it is important that we get the information directly from them (patient) [rather than making assumptions]” [UAT20, caregiver] “They are very concise, not too long, not too short, but every point that is important I guess it’s all mentioned” [UAT03, medical social worker]
	Suitable duration	✓✓✓	✓✓✓	✓✓	“The length of My Voice is just right” [UAT13, patient]
**Theme 3: usefulness in understanding the patient’s values and goals**					
	Complementary to ACP	—^d^	—	✓✓	“I think this website is in a way like helping the patient to do the first part [of ACP] to get them to think about it before they come and really start to think in detail what are the treatments they want” [UAT01, ACP facilitator] “It’s more effective to reach the mass rather than in every admission or outpatient when we refer to ACP coordinator” [UAT03, medical social worker]
	Understanding values and goals	✓✓✓	✓✓✓	✓	“It addresses the issue (by allowing the patient to let their preferences be known) especially when my husband does not want to listen … if there is an additional step that can tell me what to do when my husband doesn’t want to listen to me that will be great” [UAT25, patient] “It is really true that based on their condition, they might change. It’s not always that – and then human heart or mind may be a bit fickle, or they might change based on their condition. So, this (frequent updates) is quite a good way” [UAT20, caregiver]
	Saves time	—	—	✓	“This will cut down a lot of professional’s time and caregiver’s time [during the ACP process]” [UAT03, medical social worker]

a HCP: health care professional.

b ✓ indicates a positive response and x indicates a negative response. Number of ✓ indicates strength of the responses (✓✓✓ strong, ✓✓ moderate, and ✓ mild), and likewise for x.

c ACP: advance care planning.

d Not applicable.

**Table 3.** Participant suggestions for improving “My Voice” website.

Suggestion		Changes made to “My Voice”
**User experience of navigating the website**		
	“I think the layout for most pages it’s fine, except for those on the boxes right, with a solid background of blue or dark blue, the white fonts could be bolder, I think that would be more clear to the respondents” [UAT03, medical social worker]	Replaced red text on blue background with yellow text on blue background for better readability.
	“A little bit lengthy, especially for the elderly patients whose eyesight is not so good … The shorter the better. Otherwise, they have a lot to scroll, and read, and their attention span is already so short” [UAT10, nurse]	Added expected time to completion of the website - “This program will take approximately 30 min to complete.“ Reduced the length of on-page explanation before the value-clarification questions. Added a color coded progress bar on the pages to track completion.
**Acceptability of website content and duration**		
	“The options in the respective pages… can streamline them… can further divide them like personal, family, work, or finances” [UAT03, medical social worker]	Merged similar terms to shorten the text in responses options for the value-clarification questions (eg including “pain” under “symptoms” instead of 2 separate fields). Added explanations in brackets for terms that are not immediately understandable such as: “Make a legacy (something that is passed on, monetary or non-monetary)” and “Being cared for at home, rather than in an institution (eg, hospital).”
	“Don’t just give the bitter truth, add some element of sweetness. Give some hope” [UAT26, patient]	Rephrased explanations for answers to the knowledge quiz to be more empathetic (eg, “Heart failure is a serious condition that can shorten life. For some patients, heart failure is stable for a long time then gets worse. Others have a gradual decline over time.” → “We wish heart failure got better over time. Unfortunately, heart failure is a serious condition. For some patients, heart failure is stable for a long time then gets worse. Others have a gradual decline over time.”
	“Some thoughts about the Step 4 when it mentions about the medical records, patients may not know how to go about. They may have queries about this part, like how do I document, who do I approach” [UAT01, advance care planning facilitator]	In the patient module, integrated the step for documenting patient preferences in medical records with the step to speak to your doctor (step 4).
	“You will need to use simple words because sometimes when they age, complicated words they won’t understand” [UAT29, patient]	Replaced wordy introduction with simple bullet points for describing the steps involved. Used simpler terms (eg, “spokesperson 1” and “spokesperson 2” instead of “primary spokesperson” and “secondary spokesperson”).

### Survey Results

The overall mean SUS score of 74 (SD 14.8; range: 42.5 to 95), with mean scores in each of the three groups of participants exceeding the minimum cutoff score of 68, indicated good usability. Specifically, 70% of participants had scores above 68, which included 64% (7/11) of patients, 78% (7/9) of caregivers, and 70% (7/10) of HCPs (Table 4).

**Table 4.** System Usability Scale (SUS) scores by participant group.

	Patients (n=11)	Caregivers (n=9)	Health care professionals (n=10)	Overall (N=30)
**SUS score**				
Range (0‐100)	47.5‐92.5	50‐95	42.5‐95	42.5‐95
Mean (SD)	72.3 (15.2)	76.4 (15.3)	73.8 (15.2)	74 (14.8)
**SUS score category, n (%)**				
>87	2 (18)	2 (22)	3 (30)	7 (23)
69‐87	5 (46)	5 (56)	4 (40)	14 (47)
50‐68	3 (27)	2 (22)	2 (20)	7 (23)
<50	1 (9)	0 (0)	1 (10)	2 (7)

Patients highly rated the way information was presented in the 5 steps, with over 80% of patients rating each of the 12 items on acceptability as good or excellent. Likewise, over 88% of caregivers rated each of the 3 items about acceptability as good or excellent (Table 5). Except for one patient who found the length of the website to be “too long,” 19 of 20 patients and caregivers (95%) rated the website length to be “just right,” and 18 of 20 patients and caregivers (90%) rated the amount of information presented to be “just right.” Seven of 10 HCPs (70%) rated 11 of the 15 items as strongly agree or agree. Items that received lowest levels of agreement (agree or strongly agree) included—“My Voice website is better than how I usually go about conducting ACP” (30%), “using My Voice website does not involve making major changes to the way I usually do things” (50%), “My Voice website is compatible with the way I think things should be done” (60%), and “the use of My Voice website is more cost-effective than my usual approach to conducting ACP” (60%; Table 5)

**Table 5.** Acceptability ratings by patients (n=11), caregivers (n=9), and health care professionals (n=10).

Item	Value, n (%)
**Patients: good or excellent rating**	
About heart failure (step 1)	10 (91)
Thinking about goals for end-of-life care (step 2)	10 (91)
Understanding what is important to you when it comes to your health	11 (100)
Questions on what makes life meaningful to you	11 (100)
Questions on when it gets to my health getting worse, what worries me most	9 (82)
Questions on choosing a preferred treatment	10 (91)
Questions on what matters most to you and choosing top 3 goals	9 (82)
Ranking the order of the top 3 goals	9 (82)
Choosing a healthcare spokesperson (step 3)	9 (82)
Document what is important to you in medical record	10 (91)
Discussing with the doctor about “My Voice” document (step 4)	9 (82)
Revisiting “My Voice” document periodically (step 5)	9 (82)
**Caregivers: good or excellent rating**	
About heart failure (step 1)	8 (89)
Talking to your loved ones about goals for end-of-life care (step 2)	9 (100)
Supporting your loved ones (step 3)	8 (89)
**Healthcare professionals: agree or strongly agree**	
It will be easy for me to use “My Voice” for introducing advance care planning (ACP^a^) to my patients.	8 (80)
It is easy for me to understand “My Voice.”	9 (90)
It will be easy for me to use “My Voice” website for advocating advance care planning.	8 (80)
The results of using “My Voice” website will be easy to see (increase in self-administered ACP-My Voice document)	8 (80)
This “My Voice” website is better than how I usually go about conducting ACP	3 (30)
This “My Voice” website is compatible with the way I think things should be done.	6 (60)
The use of “My Voice” website is more cost-effective than my usual approach to conducting ACP	6 (60)
Compared with my usual approach, “My Voice” website will result in my patients making more informed decisions.	8 (80)
Using “My Voice” website will save me time.	10 (100)
This “My Voice” website is a reliable method of helping patients do an ACP.	7 (70)
Pieces or components of the “My Voice” website can be used by themselves.	7 (70)
This type of “My Voice” website is suitable for helping patients make value laden choices.	9 (90)
This “My Voice” website complements my usual approach to conducting ACP.	8 (80)
Using this “My Voice” website does not involve making major changes to the way I usually do things.	5 (50)
There is a high probability that using this “My Voice” website may cause / result in more benefit than harm.	7 (70)

a ACP: advance care planning.

## Discussion

### Principal Findings

Study results show that “My Voice” ACP website was well received by patients with heart failure, their caregivers, and HCPs. Most participants provided positive feedback regarding their experience using “My Voice.” They found the website easy to navigate, its duration and content acceptable, and valuable in conveying and comprehending patient values and goals. The mean usability score of 74 (SD 14.8) exceeded the a priori threshold (68 and above) for both overall and within each participant group. Additionally, patients and caregivers’ acceptability ratings were high for all items, and 80% (8/11) of patients and 78% (7/9) of caregivers rated the website as good or excellent, while 70% (7/10) of HCPs rated 11 of the 15 items as strongly agree or agree. Most patients and caregivers (19/20, 95%) and 90% (18/20) of patients and caregivers found the length and amount of information in “My Voice” just right. These findings confirm the usability and acceptability of “My Voice.”

The findings on usability and acceptability are consistent with those of other web-based ACP decision aids [45-47]. Our findings regarding HCPs’ views of the decision aid as a tool for initiating ACP discussions also align with previous research [48]. However, in contrast to our findings, one study reported that participants had difficulty understanding and engaging with some website content [49].

Given that a significant proportion of patients with heart failure are older with lower literacy levels [50], feedback primarily focused on enhancing the website accessibility for this demographic. Suggestions included simplifying the login process, improving the layout, reducing wordiness, increasing font size, and simplifying terminology. Moreover, some patients expressed surprise upon learning about the incurable nature of their illness, indicating that they had not engaged in serious illness conversations with their HCPs. Our previous studies have also shown similar findings [51,52].

Patients and caregivers rated “My Voice” to be both acceptable and usable. However, while, HCPs rated its usability highly, they provided lower ratings to certain aspects of acceptability. These included suggestions that “My Voice” could potentially be more effective and replace the current ACP facilitation method. It is important to note that “My Voice” is primarily a patient preparation tool intended to complement, rather than replace, the patient-HCP conversations. Therefore, the concerns raised are not unexpected given its supplementary role in the process.

While web-based ACP interventions are increasingly prevalent in the literature, our intervention is innovative in several respects. First, it incorporates structured mechanisms to encourage frequent revisits to “My Voice,” through educating patients and caregivers about its importance and sending reminders to them via phone. Our previous research revealed that ACP conversations typically occur as one-time events despite evolving patient preferences [22-25]. “My Voice” thus addresses this current gap in ACP implementation by facilitating ongoing reflection on values and goals and fostering periodic ACP conversations with HCPs. Second, it is tailored specifically to patients with heart failure, featuring educational videos and a quiz regarding their illness. This targeted education not only imparts urgency but also provides the context for patients to reflect on their values and goals. Third, “My Voice” seamlessly integrates active caregiver involvement into the ACP process. Not only do we coach patients to choose a surrogate decision maker and engage with them, but our dedicated caregiver module educates the chosen surrogate about the patient’s illness and guides them on how to communicate with the patient and the HCPs. This caregiver module also facilitates the sharing of the patient’s “My Voice document” with the surrogate. This approach enhances the caregiver’s understanding of patient values and goals, preparing them to make end-of-life decisions for their loved ones. Importantly, caregivers in our usability study appreciated the “My Voice” website’s unique features, particularly its capability for patients to periodically update their goals and share them with their surrogates. Lastly, the inclusion of content in three different languages enhances the accessibility of “My Voice” to a broader range of ethnic and language groups, promoting inclusivity and ensuring that individuals from diverse backgrounds can effectively engage in the ACP process.

“My Voice” is one of the first web-based ACP interventions for patients with heart failure in the Asian context. This study’s strength lies in its use of a mixed methods design to gather participant feedback, agile methodology implementation to enhance user satisfaction, and inclusion of different ethnic and language groups across multiple sites. However, there are some limitations. The response rate from patients was low (46%), partly attributed to the older age of many participants approached and their reluctance to engage in web-interventions. Future studies could improve response rates by involving health and social care providers, community organizations, or adult children to facilitate initial contact with older individuals. Additionally, although the sample size was adequate to achieve thematic saturation, it was too small to discern variations across age, gender, and other sociodemographics.

### Practice Implications

“My Voice” is a usable and acceptable tool for empowering patients to engage in ACP conversations with their caregivers and HCPs. The efficacy of the “My Voice” website in improving patient and caregiver outcomes is being assessed through a randomized controlled trial.

### Conclusion

The findings support the usability and acceptability of the web-based ACP intervention, “My Voice,” among patients with heart failure and their caregivers. Participants largely endorsed the interactive website as a valuable tool for communication and understanding patients’ values and goals, offering constructive feedback to enhance its user-friendliness for older patients.

## Supplementary material

10.2196/60117 Multimedia Appendix 1 Select pages from "My Voice" website.

10.2196/60117 Multimedia Appendix 2 Qualitative interview guide.

## Abbreviation

ACP: advance care planning
COM-B: capability, opportunity, motivation to enhance behaviors
HCP: health care professional
SUS: System Usability Scale
