# Supplementary material for: Development and Usability of an Advance Care Planning Website (My Voice) to Empower Patients With Heart Failure and Their Caregivers: Mixed Methods Study
Source: JMIR Aging. 2024 Dec 18;7:e60117. doi: 10.2196/60117 (PMC11669373; doi:10.2196/60117)
Supplement: Multimedia Appendix 1 [file aging-v7-e60117-s001.pdf]

My Voice

HOME ENGLISH 中文 MELAYU HELP

Welcome to My Voice

'My Voice' is a programme for people who have heart failure and their caregivers to help them discuss and prepare for their current and future medical care. The programme will take approximately 30 minutes to complete.

First-time Users

Create an Account

Already Registered Users

Sign in as Patient

Sign in as Caregiver

Use as a Guest

Home page

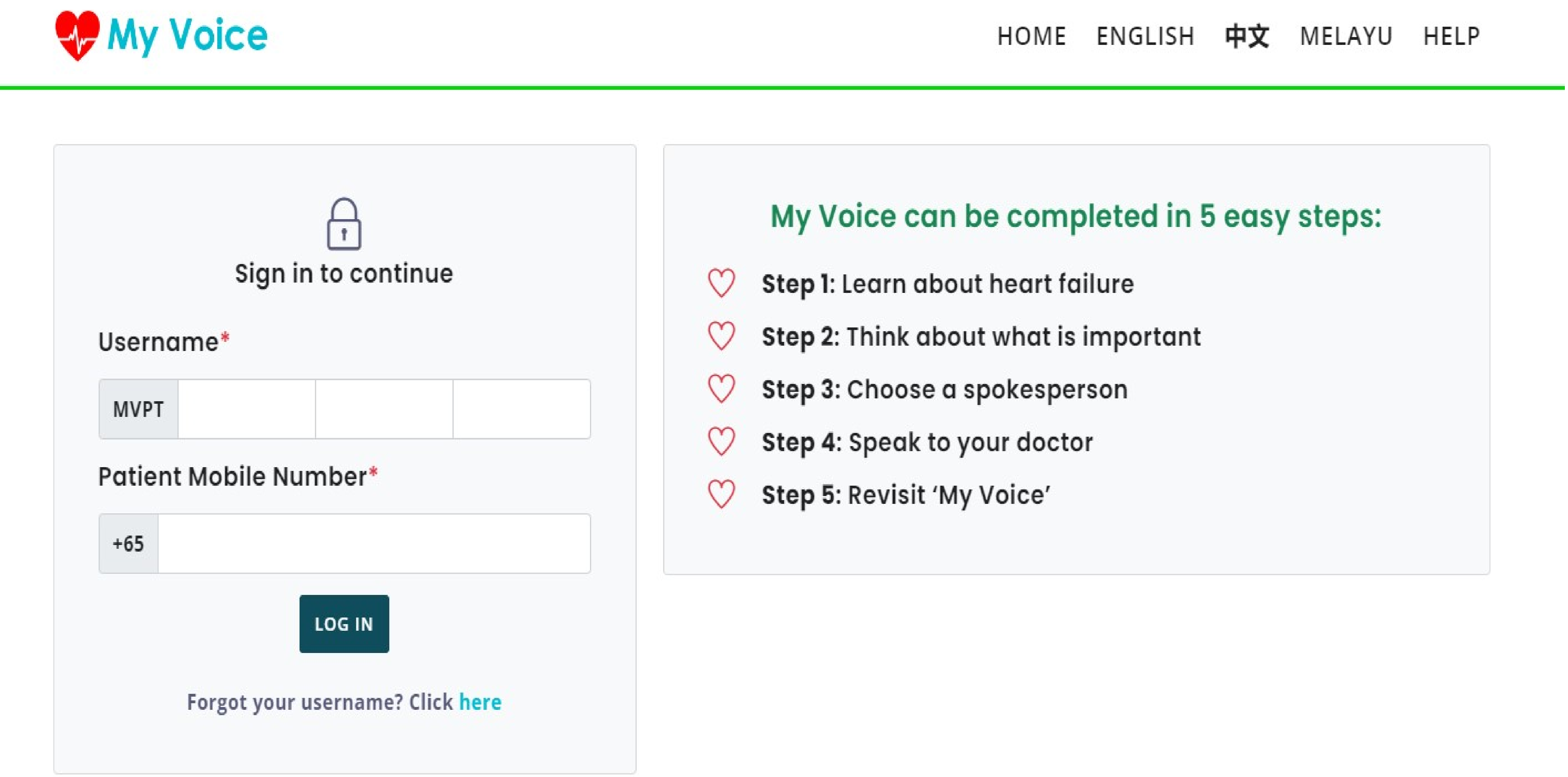

Patient module

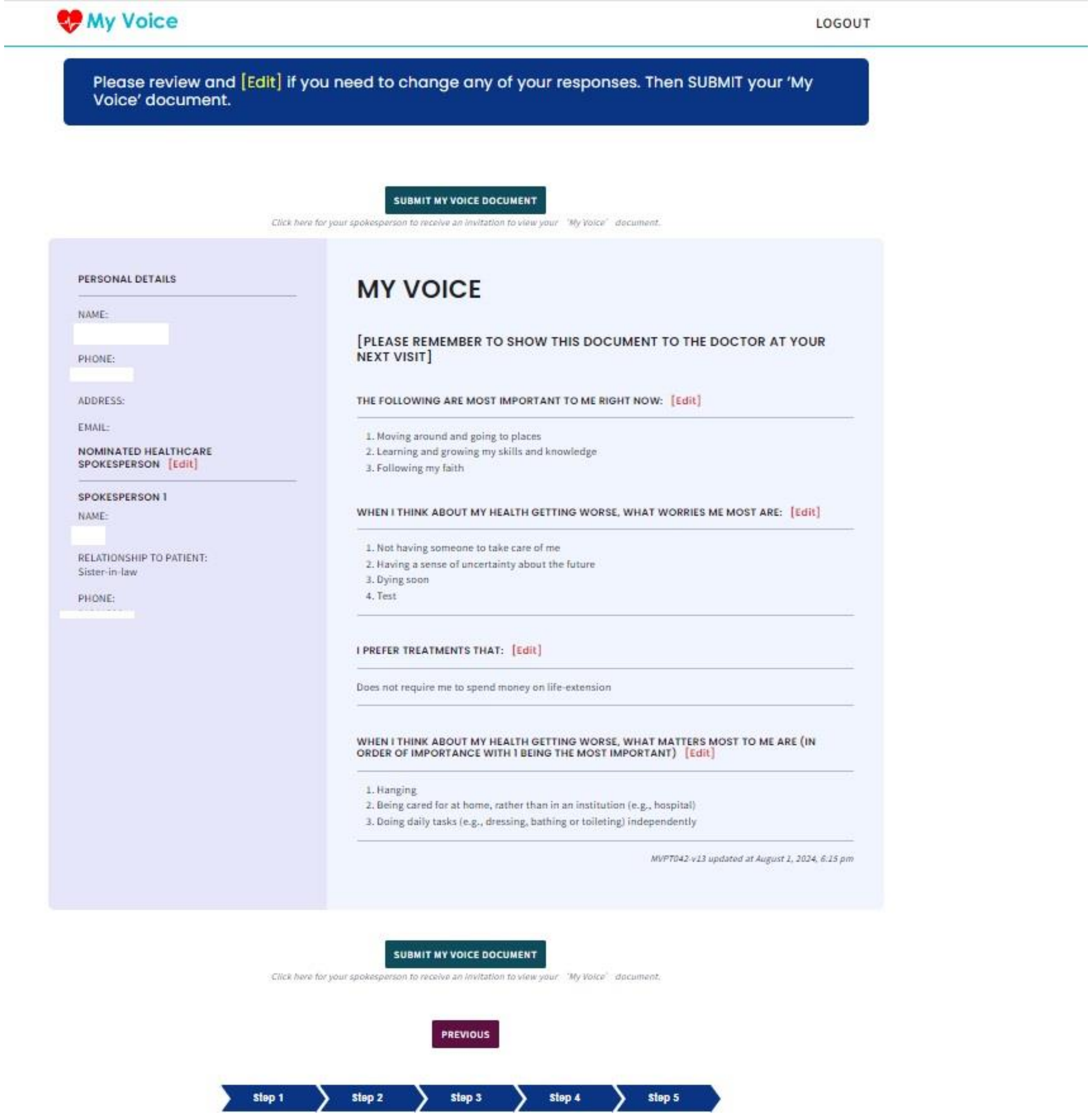

'My Voice' document
