# Supplementary material for: Development and Usability of an Advance Care Planning Website (My Voice) to Empower Patients With Heart Failure and Their Caregivers: Mixed Methods Study
Source: JMIR Aging. 2024 Dec 18;7:e60117. doi: 10.2196/60117 (PMC11669373; doi:10.2196/60117)
Supplement: Multimedia Appendix 2 [file aging-v7-e60117-s002.pdf]

Open ended questions for ‘My Voice’ usability testing for different participant groups

Patients

For each web-page ask:

A1	What were you thinking as you viewed this video/read this page?
A2	How easy or difficult was this to understand?
A3	What are your thoughts on the language used in the video/text?

For Step 2 questions, ask about each question:

A4	What did you think about this question?
A5	Were you able to choose the desired answers?<br>- If no, why?
A6	What did you like the most about using the ‘My Voice’ website?
A7	What did you like the least about using the ‘My Voice’ website?
A8	What, if anything, surprised you about using the ‘My Voice’ website?
A9	What, if anything, caused you frustration when using the ‘My Voice’ website?
A10	What did you think of the layout of the ‘My Voice’ website?
A11	What did you think of the on-page explanations?
A12	What was your overall impression of the ‘My Voice’ website?
A13	What would you like to change about the ‘My Voice’ website?
A14	Do you find this ‘My Voice’ website useful?
A15	What suggestions do you have to improve ‘My Voice’ website?
A16	Would you recommend the ‘My Voice’ website to a patient like you?

Caregivers

**For each web-page ask**:	
B1	What were you thinking as you viewed this video/read this page?
B2	How easy or difficult was this to understand?
B3	What are your thoughts on the language used in the video/text?
B4	What did you like the most about using the 'My Voice' website?
B5	What did you like the least about using the 'My Voice' website?
B6	What, if anything, surprised you about using the 'My Voice' website?
B7	What, if anything, caused you frustration when using the 'My Voice' website?
B8	What did you think of the layout of the 'My Voice' website?
B9	What did you think of the on-page explanations?
B10	What was your overall impression of the 'My Voice' website?
B11	What would you like to change about the 'My Voice' website?
B12	Do you find this 'My Voice' website useful?
B13	What suggestions do you have to improve 'My Voice' website?

Healthcare Providers

For each web-page ask:

A1	What were you thinking as you viewed this video/read this page?
A2	How easy or difficult was this to understand?
A3	What are your thoughts on the language used in the video/text?

For the patient's version, also ask the following:

For each question in Step 2:

A4	What did you think about this question?
A5	Do you think we included enough information to help patients decide about their goals for end-of-life care? Do you wish to add any?

For Step 3: Choosing a healthcare spokesperson, ask:

A6	Do you think we included enough information? Do you wish to add any?
A7	Do you think we included enough information in the 6 steps to help patients with advance care planning?

For both patient and caregiver versions also ask:

A8	What did you like the most about using the 'My Voice' website?
A9	What did you like the least about using the 'My Voice' website?
A10	What, if anything, surprised you about using the 'My Voice' website?
A11	What did you think of the layout of the 'My Voice' website?
A12	What did you think of the on-page explanations?
A13	What was your overall impression of the 'My Voice' website?
A14	What would you like to change about the 'My Voice' website?
A15	Do you find this My Voice website useful? How do you compare it with the traditional way of conducting ACP?
A16	What suggestions do you have to improve My Voice website?
A17	Do you think My Voice website will be useful for patients? Why or why not?
A18	Would you recommend the My Voice website to your patients and their caregivers? Why or why not?

.
